# The assessment of computationally derived protein ensembles in protein-ligand docking

**DOI:** 10.1186/1758-2946-4-S1-P34

**Published:** 2012-05-01

**Authors:** Barbara Sander, Oliver Korb, Jason Cole, Jonathan W Essex

**Affiliations:** 1 School of Chemistry, University of Southampton, Highfield, Southampton, SO17 1BJ, UK; 2 Cambridge Crystallographic Data Centre, 12 Union Road, Cambridge, CB2 1EZ, UK

## 

The inclusion of receptor flexibility in protein-ligand docking experiments has become a major research interest in drug discovery [1,2]. One of the possible methods applied is the use of multiple discrete protein conformations, so called ensemble docking [3,4]. With computational techniques like Molecular Dynamics (MD) a large number of different conformations can be generated, not all of which can or should be included in the docking or virtual screening process [5]. The question arises if and how suitable protein conformations can be selected systematically *a priori* based on quantifiable conformational features.

For neuraminidase and cyclin-dependent kinase II (CDK2), snapshots of MD simulation trajectories have been clustered based on different structural properties using a variety of clustering methods. To establish a possible correlation between docking performance and target conformational attributes the clustered snapshots have been subjected to extensive self- and cross-docking experiments as well as virtual screening using the GOLD docking programme. It is shown that conformationally similar snapshots do not necessarily result in a similar docking or virtual screening performance. The selection of the particular structural property on which to base the clustering appears to be the essential problem.
